# Lassar’s Method for Treating the Scalp

**Published:** 1890-02

**Authors:** Ernest Wende


### LASSAR'S METHOD FOR TREATING THE SCALP.

Translated from *Der Fortschritt*, for the BUFFALO MEDICAL AND SURGICAL JOURNAL,

BY ERNEST WENDE, M. D., B. SC.

WE HAVE no means to prevent hair from falling out nor any to hasten a new growth; consequently, treatment is naturally superfluous, especially since, in the majority of cases a complete restoration sets in spontaneously. Thus frankly remarked Lassar in the chapter on alopecia areata in his manual on skin diseases. The disease, as is well known, attacks the scalp, forms circular spots about the size of a dollar, and from the margin the hair comes out readily on slight traction. With its advance complete baldness may ensue. We may still read in the recognized compendium of Kunze that "treatment which would prove effectual is not known." Here, also, may be found the indifferent attitude of physicians regarding this affliction. Whenever a young man is seen whose baldness is conspicuous, we may hear some trivial remark ascribing the cause to excesses in "Venere et Baccho," which by the way is often a false conclusion. The sudden fall of hair is a disorder to which some (Sehlen and Unna) assign a parasitic cause, while others again, as Michelson, attribute it to a nervous origin, although the use of unclean utensils by the barber is frequently responsible for it.

The first to arouse physicians from lethargy in the treatment of alopecia was Lassar, the well-known and able docent for diseases of the skin at the University of Berlin. In an article on diseases of the hair he puts forth his method which he had tried in more than 1,000 cases of alopecia pilyrodes and areata, and gives the following directions:

*First.* The scalp must be well lathered with a very strong tar soap for ten minutes.

*Second.* The lather is removed first with luke-warm followed with colder water in abundance, after which the scalp is thoroughly dried.

*Third.* The scalp is then rubbed with the following solution:

| | |
|---|---|
| ℞—Sol. Hydrarg. bichlor. corr. . . . . . . . . . . . . . | 0.5:150.0 |
| Glycerin . . . . . . . . . . . . . . . . . . . . . | |
| Spirit. or cologn aa . . . . . . . . . . . . . . . . . . | 50.00 |
| M—Sig. Ext. | |

*Fourth.* The scalp is rubbed dry with a solution of

| | |
|---|---|
| ℞—Beta naphtholi . . . . . . . . . . . . . . . . . . . . | 0.5 |
| Absol. alcohol . . . . . . . . . . . . . . . . . . . . | 100.00 |
| Mix. | |

*Fifth.* After this, the scalp is thoroughly anointed with a liberal application of the following preparation:

| | |
|---|---|
| ℞—Acidi Salicylici . . . . . . . . . . . . . . . . . . . . | 2.00 |
| Tr. Benzoes . . . . . . . . . . . . . . . . . . . . . . . | 3.00 |
| Ol. ped. taur. q.s. ad . . . . . . . . . . . . . . . . . | 100.00 |
| Mix. | |

This procedure must be kept up for six to eight weeks, and be repeated every day.

But few cases resist the treatment, and often after a few applications the downy sprouts may be seen.

Dr. Graetzer, in the October number of the *Therap. Monatsch.*, warmly advocates this excellent method. He reports brilliant results obtained from its use, and invites his colleagues to give it a more extended trial than heretofore.

In making this reference to Lassar's method, I did not regard it as altogether purposeless, since there are so many young pharmacists and physicians who carry about barren fields upon their heads, the result of alopecia, who, perhaps, would make another attempt at cultivation.

---

E. B. TREAT, publisher, No. 5 Cooper Union, New York, announces the *International Medical Annual* for 1890, as shortly to appear. This is the eighth issue of this periodical, will contain about 600 pages, illustrated, and will be in many respects superior to its predecessors. Its corps of thirty-eight editors embraces some of the most competent medical writers of the present day, and the *Annual* is fast becoming a necessity for the active physician. Orders should be placed at once. Price, $2.75.

---

VICK'S FLORAL GUIDE for 1890, published by the veteran Rochester seedsman, whose name it bears, has appeared, and it is more complete and handsomer than ever. Every flori-culturist, and particularly all women interested in garden and house plants, should see this beautiful illustrated catalogue of the largest establishment of its kind in this country. Price, 10 cents (to be deducted from the first order), which amount sent to James Vick, seedsman, Rochester, N. Y., will secure a copy.

---

THE INTERNATIONAL CARRIAGE CO. of Buffalo, N. Y., sends out a most artistic circular as a special newspaper edition. Its pictorial trade-mark is at once an enigma and a study in art engraving.

